# Perceived Dignity of Advanced Cancer Patients and Its Relationship to Sociodemographic, Clinical, and Psychological Factors

**DOI:** 10.3389/fpsyg.2022.855704

**Published:** 2022-05-26

**Authors:** Berta Obispo, Patricia Cruz-Castellanos, Raquel Hernandez, Mireia Gil-Raga, Manuel González-Moya, Jacobo Rogado, Helena López-Ceballos, Miguel García-Carrasco, Paula Jiménez-Fonseca, Caterina Calderon

**Affiliations:** ^1^Department of Medical Oncology, Hospital Universitario Infanta Leonor, Madrid, Spain; ^2^Department of Oncology Medical, Hospital Universitario La Paz, Madrid, Spain; ^3^Department of Oncology Medical, Hospital Universitario de Canarias, Santa Cruz de Tenerife, Spain; ^4^Department of Medical Oncology, Consorci Hospital General Universitario de Valencia, Valencia, Spain; ^5^Department of Medical Oncology, Hospital Quirónsalud, Seville, Spain; ^6^Department of Medical Oncology, Hospital San Pedro de Alcántara de Cáceres, Cáceres, Spain; ^7^Department of Medical Oncology, Hospital Universitario Central de Asturias, Oviedo, Spain; ^8^Department of Clinical Psychology and Psychobiology, Faculty of Psychology, University of Barcelona, Barcelona, Spain

**Keywords:** advanced cancer, dignity, quality of life, social support, mental adjustment, psychological factors

## Abstract

**Objective:**

Loss of dignity is one of the main reasons for wishing for an early death in patients with incurable diseases such as cancer and is strongly associated with psychological distress and loss of quality of life. The present study aims to analyze the perceived dignity of patients with advanced cancer undergoing systemic treatment and their relationship with sociodemographic, clinical, and psychological factors.

**Methods:**

A prospective, cross-sectional, multicenter study was conducted in 15 oncology departments in Spain. Patients with locally advanced, unresectable, or metastatic cancer who were candidates for systemic treatment were included. Participants completed demographic information and Palliative Patients’ Dignity Scale, Brief Symptom Inventory, Mental Adjustment to Cancer, Functional Social Support Questionnaire, and Illness Uncertainty.

**Results:**

A total of 508 patients were recruited between February 2020 and October 2021. Most were male, aged > 65 years, with digestive tumors (41%), and metastatic disease at diagnosis. Subjects were classified as having low (56%, *N* = 283) or high (44%, *N* = 225) perceived dignity. Patients ≥ 65 years, with worse baseline status (ECOG ≥ 1), and worse estimated 18-month survival had lower levels of perceived dignity. People with lower perceived dignity scored higher for anxious preoccupation and hopelessness and lower for positive attitude. They also displayed higher levels of anxiety, depression, and somatic symptoms, greater uncertainty, and less social support.

**Conclusion:**

Self-perceived dignity in advancer cancer patients is significantly associated with psychological factors, psychological distress, uncertainty, less social support. Knowledge of these specific interactions is importance for adequate, comprehensive palliative care.

## Introduction

Cancer is one of the leading causes of death worldwide; its incidence has increased progressively in recent years, and the number of cases is expected to increase by 2040 (Dyba et al., 2021). Many patients perceive receiving a diagnosis of cancer and the subsequent treatment procedures as stressful, putting individuals with advanced cancer at high risk of suffering psychological problems (Hinz et al., 2010; Zhu et al., 2017; Pitman et al., 2018). Thanks to advances in anti-tumor treatments, overall survival rates are currently longer with the consequent risk of greater emotional disturbances throughout the illness. The after-effect of treatment and the course of the disease itself can alter the patients self-identity, affecting their perceived dignity. It can disrupt family and social relationships and increase symptoms of depression, anxiety, and feelings of hopelessness (Chochinov, 2004). All these factors potentially compromise their quality of life, especially in patients with advanced cancer (Hosseini et al., 2017).

The loss of dignity is one of the main reasons for wishing for an early death in people with incurable diseases such as cancer; likewise, it is one of the main reasons for requesting assisted death (Kennedy, 2016). Cancer patients, especially those with advanced disease, seek to adapt to their illness so as to prepare for their grief in the face of impending death. Recent studies reveal that this preparatory grief may be related to the perception of dignity (Adib-Hajbaghery and Aghajani, 2015). Loss of dignity among individuals with advanced cancer is associated with high levels of psychological and spiritual distress or suffering, as well as a significant decline in the will to live (Griffin-Heslin, 2005).

The importance of preserving the cancer patient’s dignity is evident, but the psychological approach to these cases is still failing at many centers. Moreover, the relationship between sense of dignity and demographic, clinical, and psychological factors has yet to be extensively studied and have been constrained by the ambiguous concept of dignity.

Although the concept of dignity is known as a universal necessity for the well-being of both the individual and society, its definition is unclear and multidimensional (Chochinov, 2002; Chochinov et al., 2008). Four concepts have been described as lying at the heart of dignity: respect (for oneself and others), autonomy (decision-making, independence), empowerment (self-esteem, pride), and communication. Each of these attributes is, in turn, multidimensional, which accounts for the complexity surrounding the concept of dignity (Rudilla et al., 2016).

Chochinov is one of the most important authors involved in the study of dignity in palliative care patients. This author was the first to develop a model capable to assess the perceived dignity terminally ill. In 2002, he developed a preliminary model, based on patient interviews, in which he described three factors that determine dignity: the “illness related concerns,” “the dignity conserving repertoire,” and “social dignity inventory.” Firstly, concerns about the disease refer to how the consequences of the illness itself may affect the dignity of the person by losing cognitive or functional independence, and suffering discomfort from physical or emotional symptoms. The “dignity conserving repertoire,” which can include conserving perspectives of dignity (autonomy, acceptance, resilience) and dignity-preserving practices (living in the moment, seeking spiritual comfort). Finally “social dignity inventory” includes issues that influence patients’ sense of dignity such as: privacy barriers due to the need for more care, lack of social support, the attitude of others when interacting with the patient, the feeling of being a burden to others and concerns about what will happen after their death (Monforte-Royo et al., 2012; Kostopoulou et al., 2018). From this model, a reliable and validated questionary, the Patient Inventory Dignity (PDI), was derived, which assesses dignity-related distress in several dimensions. Years later and building on this model, Rudilla et al. developed the Palliative Patients’ Dignity Scale (PPDS), a new, brief instrument to assess dignity based on the perceptions of patients, relatives, and professionals (Hall et al., 2009). Dignity was defined in the context of four categories: dignity as an attitude, as an intrinsic quality, as a right and as well-being or hedonism. The brevity of the questionnaire, with only eight items, makes it ideal to be applied in the clinical context.

Most studies on dignity in cancer patients have been carried out in the terminal phase, but we have no data on the patient’s sense of dignity when diagnosed with an incurable but treatable disease and the factors that may be related. Moreover, as we have previously described, the survival of patients with incurable cancer is increasing, so it seems consistent to think that an early assessment of dignity from the diagnosis of advanced disease together with a correct approach could contribute to improve or preserve it, reducing psychological distress throughout the evolution of cancer, contributing to psychological well-being and increasing quality of life. Further study of dignity in the cancer population is needed to understand with certainty what factors are potentially related to their sense of dignity and to identify those subgroups of patients who may benefit most from early intervention strategies (Chochinov et al., 2005, 2011; Xiao et al., 2019).

The present study aims to analyze the perceived dignity of people with advanced cancer undergoing systemic treatment in Spain and its correlation with sociodemographic, clinical, and psychological factors.

## Materials and Methods

### Participants and Procedures

This was a multicenter, prospective (data collection chronology), cross-sectional (design) study. A consecutive sample of advanced cancer patients was recruited at 15 medical oncology departments at different hospitals in Spain, between February 2020 and October 2021. Patients were selected at their first visit to the medical oncologist during which their diagnosis, stage of disease, incurable disease status, and systemic antineoplastic treatment options were explained. Eligible patients were ≥18 years with histologically confirmed advanced cancer who were not eligible for surgery or other therapy with curative intent. Individuals were excluded if they had a physical condition, comorbidity, and/or age that contraindicated antineoplastic treatment in the attending oncologist’s opinion; those who had received cancer treatment in the previous 2 years for another advanced cancer, or with any underlying personal, family, sociological, and/or medical condition that could hinder their ability to participate in the study were excluded (those with cognitive impairment or severe deterioration of general status due to cancer or other causes that prevent them from understanding and reasoning what is asked in the questionnaires). This research was conducted in accordance with current ethical principles and received prior approval from the Ethics Review Committees at each institution and from the Spanish Agency of Medicines and Health Products (AEMPS; identification code: ES14042015). The study comprised the completion of several questionnaires and collection of clinical data from the interview and medical records. Data collection procedures were similar at all hospitals and data relating to the participants were obtained from the institutions where they received treatment. Participation was voluntary, anonymous, and in no way affected patient care. All participants signed informed consent prior to inclusion which is delivered by the medical oncologist. Data were collected and updated by the medical oncologist, through a web-based platform.^1^ We screened 565 patients; 508 were eligible for this analysis, and 57 were excluded (10 did not meet the inclusion criteria; 9 met at least one exclusion criterion, and 38 had incomplete data at the time of analysis).

### Measures

Demographic information including age, sex, marital status, having children, educational level, and the questionnaires were reported in writing by the patients. The five questionnaires (PPDS, BSI, Mini-MAC, UNC, IUS) were completed by the participants at home during the interval between the first visit to the oncologist and the start of systemic treatment. Clinical variables related to cancer, antineoplastic treatment, and outcomes as estimated survival, were collected by the medical oncologist from the medical records.

Dignity was assessed by the Palliative Patients’ Dignity Scale (PPDS). This instrument consists of eight items, that assesses the preservation of dignity by defining it as respect for others, for oneself and the individual’s right to decide in peace how he or she wants to be treated. On the other hand, the threat or loss of dignity is evaluated as insecurity and violation of one’s own values, as well as personal exhaustion and depletion of external social support. Items are scored on a 9-point Likert scale, with the total score ranging from 0 to 72; the higher the score, the greater the perceived dignity. The Spanish version was published by Rudilla et al. (2016). Cronbach’s alpha was 0.75 (Hall et al., 2009).

Brief Symptom Inventory (BSI) is one of the most widely used instruments to assess anxiety and depression in clinical cases (Derogatis, 1993). The questionnaire consists of 18 descriptions of physical and emotional complaints divided into three dimensions (anxiety, depression, somatization). The anxiety subscale evaluates symptoms of nervousness, tension, motor restlessness, apprehension, and panic states, while the depression subscale quantifies symptoms of disaffection and dysphoric mood, e.g., those reflecting self-deprecation, anhedonia, hopelessness, and suicidal ideation. Each item is scored on a 5-point Likert scale and the score for each subscale ranges from 0 to 24 with higher scores indicating greater anxiety or depression. Raw scores are converted to T-scores based on gender-specific normative data. To identify individuals with significant levels of anxiety and depression, the BSI applies the clinical case-rule. The Spanish version of the BSI has proven good reliability and validity in Spanish patients (Calderon et al., 2020). Cronbach’s alpha scores for the scales ranged between 0.75 and 0.88.

Coping strategies for cancer were assessed using the Mini-Mental Adjustment to Cancer (Mini-MAC) (Watson et al., 1994). This scale contains 28 items that evaluate five factors: fighting spirit, hopelessness, anxious preoccupation, cognitive avoidance, and fatalism. The items are answered on a 4-point Likert scale. The higher the score on the subscale, the greater the use of that coping strategy. The Spanish version of the Mini-MAC scores had reliability estimates (Cronbach’s alpha) ranging from 0.88 to 0.9 (Calderon et al., 2021).

The Duke/UNC Functional Social Support Questionnaire (DUFSSQ) (Broadhead et al., 1988) is a questionnaire consisting of 11 items assessing instrumental social support (receiving information, advice, or guidance) and affective social support (expressions of love, appreciation, sympathy for the patient). Social support has proven to be a key mediating variable in the stress suffered by people with cancer. Each item allows for five responses on a Likert scale. The score can therefore range from 11 to 55 with higher scores indicating less support. The scale was validated in the Spanish population (Ayala et al., 2012). Cronbach’s alpha was 0.93 (Broadhead et al., 1988).

Uncertainty was appraised using the 5-item Mishel Uncertainty in Illness Scale (MUIS) (Mishel, 1990). This questionnaire probes reactions to uncertainty, ambiguous situations, and the future. Items are scored on a Likert scale ranging from 1 to 5, yielding possible scores of 5–25; higher scores signify more uncertainty. The scale was validated in the Spanish population (Ruymán Brito-Brito et al., 2018). The Cronbach’s alpha was 0.84 (Mishel, 1990).

### Statistical Analysis

Descriptive statistics and frequency distributions were calculated for demographic and clinical characteristics using SPSS version 23 (IBM SPSS Statistics for Windows, Armonk, NY, United States: IBM Corp.). To identify patients with similar dignity patterns, a cluster analysis was conducted. Clustering variables comprised the PPDS items. Since clustering requires valid values for all variables, subjects with any missing PPDS values were eliminated. A final sample of *n* = 508 was used for the cluster analysis. We carried out a k-means method using Euclidean distances between observations to estimate clusters and Ward’s hierarchical clustering method (Ward, 1963), where the distance between two clusters is defined as the squared error criterion. In all instances, the distances were computed from the raw data to incorporate the elevation, scatter, and shape of the subject’s profiles (Cronbach and Gleser, 1953; Jaccard and Jacoby, 2019). A two-cluster solution was found to distinguish between low and high dignity. Analyses of variance (ANOVA), as well as Chi-square analyses were carried out to evaluate differences in demographic, clinical, and psychological characteristics among the dignity profiles. Bonferroni correction was used for *post-hoc* contrast. Eta squared (η^2^) were applied to assess effect size in continuous variables. Eta-squared ranges between 0 and 1, with η^2^∼0.01 for a small, η^2^∼0.06 for a medium, and η^2^ > 0.14 for a large effect size (Piercem et al., 2004). A *p-*value of < 0.05 was deemed statistically significant.

## Results

### Sociodemographic and Clinical Characteristics

At the time of data cutoff, a total of 508 patients had been recruited. Most of the individuals included were male (54 vs. 46%), aged ≥ 65 years (53 vs. 47%), married or partnered (66 vs. 13%), and had children (84 vs. 16%). The most common tumor sites were digestive (41%), bronchopulmonary (29%), breast (9%), and most had metastatic disease at diagnosis (80 vs. 20%). The most frequent treatment was chemotherapy (56%), followed by immunotherapy (7%) and targeted therapies (6%): The majority of the participants had an ECOG (Eastern Cooperative Oncology Group) status of ≥ 1 at diagnosis (66%), with an estimated 18-month survival in more than half of the cases (51 vs. 49%).

### Dignity Profiles and Clinical-Demographic Characteristics

We carried out a k-means method using Euclidean distances between observations to estimate clusters and Ward’s hierarchical clustering method. A two-cluster solution was found to distinguish between low and high dignity. After completing the PPDS scale and performing cluster analysis, subjects were classified as having low levels of perceived dignity (56%, *n* = 283) or high levels of perceived dignity (44%, *n* = 225). Analyzing the relationship between perceived dignity and clinical and demographic characteristics of the study population, we found that individuals ≥ 65 years of age (*X*^2^ = 6.718, *p* = 0.010), with worse baseline status (ECOG ≥ 1) (*X*^2^ = 11.393, *p* = 0.001), and worse estimated 18-month survival (*X*^2^ = 6.118, *p* = 0.013) correlated with lower levels of perceived dignity. No statistically significant relationship was detected with sex, marital status, educational level, having children, tumor site, histology, stage, or treatment modality (Table 1).

**TABLE 1:** Differences in demographic and clinical characteristics among the dignity profiles (*n* = 508).

Variables	Total sample n (%) 508 (100%)	Low dignity n (%) 283 (56%)	High dignity n (%) 225 (44%)	*X* ^2^	*P*-value
**Sex**					
Male	274 (54)	148 (52)	126 (56)	0.692	0.406
Female	234 (46)	135 (48)	99 (44)		
**Age**					
<65 years	236 (47)	117 (41)	119 (53)	**6.718**	**0.010**
≥65 years	272 (53)	166 (59)	106 (47)		
**Marital status**					
Married or partnered	388 (66)	202 (81)	186 (86)	2.005	0.157
No partnered	120 (13)	68 (19)	52 (14)		
**Educational level**					
Primary	243 (48)	143 (51)	100 (44)		
High school or more	265 (52)	140 (49)	125 (56)	1.860	0.173
**Having children**					
With children	425 (84)	236 (83)	189 (84)	0.034	0.854
Without children	83 (16)	47 (17)	36 (16)		
**Tumor site**					
Bronco-pulmonar	147 (29)	81 (29)	66 (29)	4.423	0.219
Digestive	207 (41)	123 (44)	84 (37)		
Breast	43 (9)	26 (9)	17 (8)		
Others	111 (22)	53 (19)	58 (26)		
**Histology**					
Adenocarcinoma	320 (63)	185 (65)	135 (60)	1.551	0.213
Others	188 (37)	98 (35)	90 (40)		
**Stage**					
Locally advanced	103 (20)	55 (19)	48 (21)	0.280	0.597
Metastatic disease (IV)	405 (80)	228 (81)	177/79)		
**Type of treatment**					
Chemoterapy	283 (56)	165 (58)	118 (52)	8.393	0.078
Immotherapy	34 (7)	12 (4)	22 (10)		
Targeted therapies	30 (6)	14 (5)	16 (7)		
Others	161 (32)	92 (33)	69 (31)		
**ECOG performance status**					
0	174 (34)	79 (28)	95 (42)	**11.393**	**0.001**
1 or more	334 (66)	204 (72)	130 (58)		
**Estimated survival**					
<18 months	248 (49)	152 (54)	96 (43)	**6.118**	**0.013**
≥18 months	260 (51)	131 (46)	129 (57)		

*Bold values indicate the significant at 5% level.*

### Dignity Profiles and Psychosocial Characteristics

When looking for relationships between dignity profiles and the psychosocial symptoms assessed by the scales (BSI, Mini-MAC, DUFSSQ, MUIS), we found that cancer patients with lower perceived dignity scored higher on anxious preoccupation (*M* = 51.6 vs. *M* = 45.6; η^2^ = 0.013) and hopelessness (*M* = 30.4 vs. *M* = 18.8; η^2^ = 0.060) and lower on positive attitude (*M* = 77.0 vs. *M* = 84.7; η^2^ = 0.028). In addition, patients with lower perceived dignity scored also displayed higher levels of anxiety (*M* = 66.0 vs. *M* = 62.1; η^2^ = 0.009), depression (*M* = 64.7 vs. *M* = 60.2; η^2^ = 0.102), somatic symptoms (*M* = 66.3 vs. *M* = 63; η^2^ = 0.004), greater uncertainty (*M* = 15.1 vs. *M* = 13.8; η^2^ = 0.022), and less social support (*M* = 41.3 vs. *M* = 45.1; η^2^ = 0.021). Conversely, those with greater perceived dignity scored lower on anxious preoccupation and hopelessness and higher on positive attitude, with lower levels of anxiety and depression, fewer somatic symptoms, less uncertainty, and more social support. No statistically significant differences were found regarding cognitive avoidance (Table 2).

**TABLE 2:** Differences in baseline psychosocial characteristics and dignity profiles.

	Low dignity (*n* = 283)		High dignity (*n* = 225)				
	Mean	SD	Mean	SD	*F*	*p*	Eta-squared
**Coping (Mini-MAC)**							
Anxious preoccupation	51.6	21.4	45.6	30.1	6.822	**0.009**	**0.013**
Hopelessness	30.4	23.6	18.8	21.9	32.057	**0.001**	**0.060**
Positive aptitude	77.0	20.9	84.7	24.2	14.380	**0.001**	**0.028**
Avoidance	62.7	25.5	64.9	25.1	0.979	0.324	–
**Psychological distress (BSI)**							
Depression	64.7	7.3	60.2	5.6	57.010	**0.001**	**0.102**
Anxiety	66.0	8.5	62.1	7.1	30.488	**0.001**	**0.009**
Somatization	66.3	7.8	63.0	7.3	22.035	**0.001**	**0.004**
Illness uncertainty (MUIS)	15.1	4.1	13.8	4.4	11.303	**0.001**	**0.022**
Social support (DUFSSQ)	41.3	9.3	45.1	19.8	10.756	**0.001**	**0.021**

*Mini-MAC, Mental Adjustment to Cancer; BSI, Brief Symptom Inventory; MUIS, Mishel Uncertainty in Illness Scale; DUFSSQ, Duke-UNC Functional Social Support Questionnaire; SD, standard deviation. Bold values indicate the significant at 5% level.*

## Discussion

After reviewing the literature, we can conclude that this study has the most patients with a recent diagnosis of advanced cancer to date, performed with the aim of exploring levels of perceived dignity in cancer patients and their relationship with demographic, clinical, and psychological factors.

In our series, we have found that more than half of the sample have low levels of perceived dignity as assessed by the PPDS (56%, *N* = 283). These results coincide with other studies in oncology reported in the literature. A 2014 German study of 61 cancer patients with advanced cancer was published that analyzed dignity using the PDI scale that yielded an average total score of 51.6 and an average of 8.7 problems (Oechsle et al., 2014). Years later, Wang conducted another study to examine the perception of dignity in mixed cancer patients. This study included a total of 202 subjects diagnosed with early and advanced cancer and found that most (89%) perceived some level of loss of dignity (mild, 71%; moderate 18%, and severe, 5%), with an average total score of 42.0 and an average of 4.5 problems on the PDI (Wang et al., 2019). In addition, this study found that the levels of the perceived loss of dignity differed between individuals with early-stage cancer and those with advanced cancer, detecting greater perceived loss of dignity in participants with advanced cancer reaching statistical significance. Similarly, a study in Germany by Vehling and Mehnert of cancer patients with early and advanced disease showed that the average total PDI score was 42.1 and the average number of problems was 4.7 (Vehling and Mehnert, 2014). All of these results are consistent with the findings of our study that included a population with advanced disease exclusively. Patients in advanced stages of cancer often lack specific treatments, which leads to intense, changing, and multifactorial symptoms associated with anxious-depressive symptoms, physical weakness, and a loss of autonomy, which could lead a loss of a sense dignity (Javaloyes Bernácer et al., 2014).

Older people are at greater risk for suffering from a perceived loss of dignity through loss of autonomy and their ability to make decisions about their own lives. With aging comes a gradual decline in physical abilities and increased dependence on others for basic activities of daily living. Cognitive deterioration sets in and patients progressively lose their ability to make decisions. In addition, health conditions are becoming increasingly complex with the emergence of new co-morbidities that make patients more fragile and more vulnerable to potentially life-threatening diseases. Moreover, age discrimination as a form of aggression (as maybe the case when choosing a cancer treatment based on age is associated with this loss of dignity (Ribera Casado, 2015). All these problems are worsened in the context of tumor disease, particularly in advanced cancer, where multiple therapeutic options may be offered but many are excluded simply because of age. Our study revealed a statistically significant relationship between low levels of perceived dignity and age ≥ 65 years, which is consistent with what we know up to the present time. Pergolizzi recently conducted a study to assess whether there were differences in the perception of dignity among advanced cancer patients according to age. The researchers included 194 participants who were included into <65 years of age and ≥65. They found that the total scores of older patients were 2.6% lower for perceived dignity-related distress compared to younger patients. These results point toward aging as a protective factor against loss of perceived dignity, contrary to what our study found with a larger population that revealed lower perceived dignity score among older individuals which could be due to loss of independence and autonomy and greater frailty (Pergolizzi et al., 2021).

We found that individuals with a worse baseline status (ECOG ≥ 1) and worst 18-month survival estimate had lower levels of perceived dignity. The physician’s assessment of the patient’s baseline functional status correlated closely with the patients’ perception of dependency. A self-perceived clinical worsening was associated with a greater sense of dependency and a greater existential distress affecting the perception of dignity, with lower values (Oechsle et al., 2014). On the other hand, it is important to note that no similar studies have been found in the literature that examine dignity measures based on estimated survival at the time of diagnosis of advanced cancer and prior to initiating treatment. Most of the studies conducted over the years on dignity are on terminally ill patients. In the previously cited study by Wang, dignity measures were analyzed in relation to time since cancer diagnosis and detected no statistically significant relationship (Wang et al., 2019). The results of our study might be accounted for by the fact that a lower estimate of survival under 18 months, is associated with clinical decline, changes in the person’s social environment, loss of autonomy, emotional changes having to do with the fear of imminent death that can contribute to a loss of perceived dignity. Therefore, we could conclude from our work that the estimation of survival to diagnosis by a medical oncologist is one of the main risk factors for the loss of dignity.

Our research detected no statistically significant relationship between gender and levels of perceived dignity. Earlier studies in cancer patients have revealed differences in dignity profiles according to gender, with females being more likely to have low levels of perceived dignity (Wang et al., 2019). This may be related to the fact that women with cancer suffer different types of distress compared their male counterparts: they suffer more from appearance-related symptoms and distress resulting from chemotherapy than men (Nozawa et al., 2013; Koyama et al., 2016). Young women with advanced cancer are particularly vulnerable to distress disorders, as they see their expected life roles and responsibilities change, usually with respect to their family and work environment, contemplating the loss of their future due to early mortality which is associated with low levels of perceived dignity (Dunn and Steginga, 2000; Park et al., 2018). The most common cancer in women is breast cancer; however, in our series it was the third most frequent tumor after digestive and lung, which could explain why no statistically significant relationship with gender was found.

Finally, when analyzing the relationship between dignity profiles and psychosocial symptoms, we found that low levels of perceived dignity are associated with high levels of anxiety, depression, and somatization (BSI scale), greater uncertainty (MUIS), and less social support (DUFSSQ questionnaire), similar to findings in previous investigations (Oechsle et al., 2014; Kostopoulou et al., 2018). Kostopoulu conducted a study in 120 patients with advanced cancer and analyzed the relationship between levels of perceived dignity and anxiety and depression assessed by HADS. The authors concluded that emotional distress has a significant association with the perception of dignity. Likewise, individuals with advanced cancer continuously live with uncertainty, which entails that both the patient and their relatives must adapt to the duration and quality of survival. This affects employment and social functions and responsibilities, leaving psychosocial sequelae that can impact their perception of dignity (Shilling et al., 2017). From a social point of view, discrepancies surrounding the cancer patient’s needs and autonomy, as a consequence of an unfair social interaction, can undermine their sense of dignity (Philipp et al., 2016).

Coping styles, especially in cancer patients facing an incurable disease, are an essential resource to mitigate the emotional stress of diagnosis and the fear of disease progression. Dysfunctional coping strategies may be related to loss of sense of dignity. Our study identified low levels of perceived dignity as correlating with higher anxious preoccupation and hopelessness scores, and lower positive attitude scores (Mini-MAC). Earlier studies have reported dysfunctional coping mechanisms in cancer patients and their association with levels of perceived dignity. Grassi developed a work that included 194 patients with early and advanced cancer. They analyzed the relationship between dignity (assessed by the PDI) and coping (assessed by the Mini-MAC HH) and found a significant association with low levels of perceived dignity and hopelessness, which is in line with the results of our study (Grassi et al., 2017).

The findings of this study must be considered in conjunction with its limitations. First, it is cross-sectional; consequently, it was not possible to determine the directionality of the relationships observed. The findings from the current study must be confirmed by longitudinal cohort studies in the future. Second, we used self-report instruments, which can lead to response bias, such as social desirability, memory errors, etc. Finally, our results cannot be extrapolated to patients with resectable cancer.

This study yields results that have clear clinical implications for the management of our cancer patients. People with newly diagnosed advanced cancer, who have not yet started any systemic treatment have low levels of perceived dignity after receiving the news of their disease, especially those ≥65 years of age, with worse performance status, and worse estimated survival. These low levels of perceived dignity denote emotional suffering for the patient with the emergence of multiple psychological symptoms. In recent years, the psychological approach to cancer patients has intensified, although most studies have been carried out in terminally ill patients. Our study has shown that addressing the person’s sense of dignity from the time of diagnosis could be the key to the multidisciplinary management of these patients, by preserving or enhancing dignity contributing to their well-being. In addition, the incidence of cancer is increasing annually, so we are faced with an increase in the number of cancer patients and an increase in survival thanks to the progressive emergence of new treatments, which highlights the importance of a psychological approach in this subgroup of patients.

In recent years, several groups have focused on interventions that could ensure the preservation of dignity through the application of dignity therapy interventions, but it is not yet very clear how this can be done and how best to measure the results. These interventions are carried out through interviews with patients in which they enrich their sense of life, reinforce their dignity, and try to alleviate psychological distress (Li et al., 2020; Nunziante et al., 2021).

This study therefore reiterates the importance of a psychological approach to advanced cancer patients and the relevance of assessing their perceived dignity, one of the pillars of fairness, equality and freedom, in order to develop strategies that improve or preserve it to improve their quality of life.

## Data Availability Statement

The data analyzed in this study is subject to the following licenses/restrictions: This database is available through a centralized web platform: www.neoetic.es. The code is available upon request to the authors. Requests to access these datasets should be directed to www.neoetic.es.

## Ethics Statement

This study was approved by the Research Ethics Committee of the Principality of Asturias (May 17, 2019) and by the Spanish Agency of Medicines and Medical Devices (AEMPS) (Identification code: L34LM-MM2GH-Y925U-RJDHQ). The study has been performed in accordance with the ethical standards of the 1964 Declaration of Helsinki and its later amendments. This study is an observational, non-interventionist trial. The patients/participants provided their written informed consent to participate in this study.

## Author Contributions

BO, CC, and PJ-F developed the project, analyzed the data, and drafted the manuscript. PC-C, RH, MG-R, MG-M, JR, HL-C, and MG-C recruited patients and provided clinical information, comments, and improvements to the manuscript. All authors participated in the interpretation and discussion of data and the critical review of the manuscript.

## Conflict of Interest

The authors declare that the research was conducted in the absence of any commercial or financial relationships that could be construed as a potential conflict of interest.

## Publisher’s Note

All claims expressed in this article are solely those of the authors and do not necessarily represent those of their affiliated organizations, or those of the publisher, the editors and the reviewers. Any product that may be evaluated in this article, or claim that may be made by its manufacturer, is not guaranteed or endorsed by the publisher.

## Footnotes

1
www.neoetic.es
